# Fatal Nonhepatic Hyperammonemia in ICU Setting: A Rare but Serious Complication following Bariatric Surgery

**DOI:** 10.1155/2016/8531591

**Published:** 2016-04-10

**Authors:** Gyanendra Acharya, Sunil Mehra, Ronakkumar Patel, Simona Frunza-Stefan, Harmanjot Kaur

**Affiliations:** ^1^Department of Internal Medicine, Wyckoff Heights Medical Center, Brooklyn, NY 11237, USA; ^2^Division of Pulmonary and Critical Care Medicine, Department of Internal Medicine, Wyckoff Heights Medical Center, Brooklyn, NY 11237, USA; ^3^Department of Medical Education, Wyckoff Heights Medical Center, Brooklyn, NY 11237, USA

## Abstract

Bariatric surgery is well established in reducing weight and improving the obesity-associated morbidity and mortality. Hyperammonemic encephalopathy following bariatric surgery is rare but highly fatal if not diagnosed in time and managed aggressively. Both macro- and micronutrients deficiencies play a role. A 42-year-old Hispanic female with a history of Roux-en-Y Gastric Bypass Procedure was brought to ED for progressive altered mental status. Physical exam was remarkable for drowsiness with Glasgow Coma Scale 11, ascites, and bilateral pedal edema. Labs showed elevated ammonia, low hemoglobin, low serum prealbumin, albumin, HDL, and positive toxicology. She remained obtunded despite the treatment with Narcan and flumazenil and the serum ammonia level fluctuated despite standard treatment with lactulose and rifaximin. Laboratory investigations helped to elucidate the etiology of the hyperammonemia most likely secondary to unmasking the functional deficiency of the urea cycle enzymes. Hyperammonemia in the context of normal liver function tests becomes diagnostically challenging for physicians. Severe hyperammonemia is highly fatal. Early diagnosis and aggressive treatment can alter the prognosis favorably.

## 1. Introduction

Bariatric surgery is well established in reducing weight and improving obesity-associated morbidity and mortality. Neurological complication such as hyperammonemic encephalopathy following bariatric surgery is rare but highly fatal if not diagnosed and treated aggressively on time. Both macro- and micronutrients deficiencies seem to play an important role in unmasking the functional deficiency of urea cycle enzymes in an adult woman after bariatric surgery.

## 2. Case Presentation

A 42-year-old Hispanic female was brought to ED with complaint of progressive altered mental status over the past few days. At ED, the patient was only responsive to painful stimuli but did not appear in acute distress. Her mother, who provided the history, had a conversation with patient three hours prior to the presentation to ED. Patient had decreased oral food intake for the last two weeks. She denied history of fever or illicit drug use except for prescribed medications. Patient had Roux-en-Y Gastric Bypass Surgery (RYGBS) two years ago for morbid obesity and gastrojejunal stent placement procedure two weeks prior to the presentation. Her medications were oral vitamins, dilaudid 4 mg, amitriptyline, and zolpidem.

**Figure 1:**
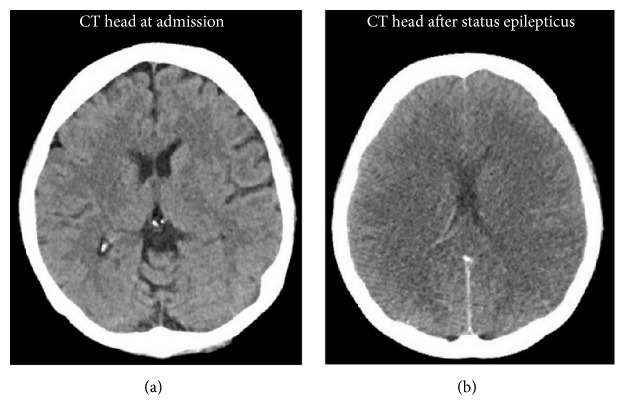
Computed tomography of the head at admission and at onset of status epilepticus. Computed tomography (CT) of the head (a) showed no remarkable findings on the day of admission and (b) showed diffused brain swelling consistent with cerebral edema on 12th day.

Physical examinations were remarkable for drowsiness with Glasgow Coma Scale (GCS) 11, ascites, and bilateral pitting pedal edema. Vital signs were within normal range. Initial labs (Table 1) were remarkable for hemoglobin (5.4 gm%), INR (2.15), aPTT (67.5 sec), BUN (11 mg/dL), creatinine (2.1 mg/dL), AST (49 IU/L) and ALT (23 IU/L), CPK (466 IU/L), low HDL cholesterol (<5 mg/dL), and prealbumin (<5 *μ*g/dL) and albumin (1.8 g/dL), and arterial blood gas (ABG) demonstrated PH 7.39. Computed tomography (CT) of the head was unremarkable (Figure 1(a)). EKG shows sinus rhythm. She was empirically treated with Narcan and flumazenil with an impression of prescription drugs overdose as urine toxicology was positive for opiates, benzodiazepines, and Tricyclic Antidepressant (TCA), but she did not improve and remained obtunded. Subsequently, she was intubated to protect her airway. Further investigations revealed elevated ammonia level (193 *μ*mol/L), low zinc level, normal vitamin B12 and folate level, negative immune and viral hepatitis panel, undetectable valproate and lithium level (Table 1), and mild hepatomegaly with fatty infiltration on CT of the abdomen. Blood and urine cultures were negative. Ascitic fluid analysis was negative for spontaneous bacterial peritonitis (SBP).

**Figure 2:**
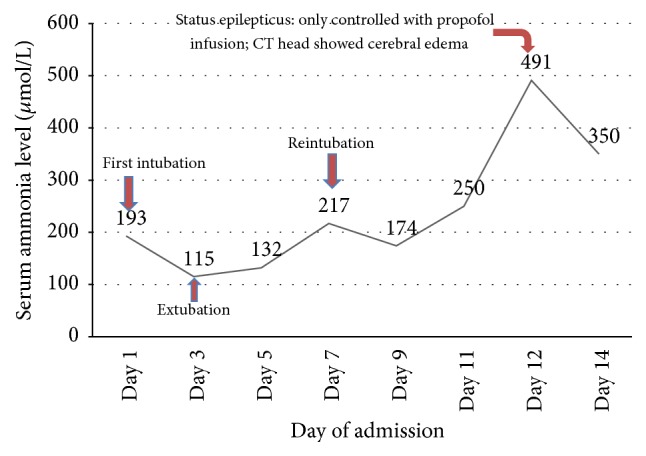
Graphical representation of serum ammonia level and associated events. Fluctuation of serum ammonia level during ICU course on this patient. Level of ammonia and mental status did not improve much with conventional treatment of hyperammonemia. The patient showed status epilepticus, which did not improve with midazolam and phenytoin and needed propofol drip to control the seizure. CT of the brain at that stage showed diffused cerebral edema.

She received two pints of packed red blood cells, IV pantoprazole, D5% fluid, lactulose/rifaximin via nasogastric tube, and vitamin K subcutaneous. Her mentation and serum ammonia (115 *μ*mol/L) improved over the next day and she was extubated. But, over the next few days, she continued to have fluctuating mental status (in and out of confusion and delirium) and serum ammonia level (Figure 2). Blood hemoglobin remained stable after the first transfusion. Coagulopathy was corrected after vitamin K supplement. Despite standard treatment for hyperammonemia, her clinical condition deteriorated and she was reintubated.

At this stage, we considered alternative explanation and conducted plasma amino acid profile and urinary orotic acid level (Table 2) to explain the etiology of intractable hyperammonemia. On the 12th day of admission, she developed status epilepticus, which was controlled only with propofol infusion. Repeat CT of the head was consistent with diffuse cerebral edema (Figure 1(b)). Before the results of amino acid profile and urinary orotic acid level came back, she rapidly deteriorated clinically over the short period of time from status epilepticus and coma to multiorgan failure and subsequently died. With elevated urinary orotic acid and serum ornithine levels, normal/low-normal serum arginine and citrulline levels along with other findings, we concluded that her hyperammonemia might have resulted in unmasking of functional deficiency of urea cycle enzyme/s in this malnourished woman following bariatric surgery. With rapid clinical deterioration followed by death, confirmatory enzymes assay and DNA testing could not be done.

## 3. Discussion

**Figure 3:**
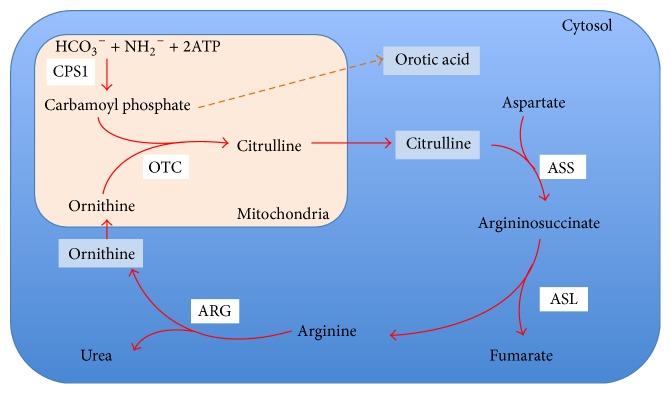
Schematic diagram of urea cycle and associated enzymes, CPS1: carbamoyl phosphate synthase 1; OTC: ornithine transcarbamylase; ARG: arginase; ASS: argininosuccinate synthetase; ASL: argininosuccinate lyase; ATP: adenosine triphosphate.

Hyperammonemia is defined as an increase in the level of ammonia in the blood. Ammonia is a toxic by-product of protein and energy metabolism through biochemical transamination and deamination process in all body tissue. Ammonia is converted to urea (ureagenesis) via urea cycle (Figure 3) primarily in hepatocytes and is excreted through the kidneys and large intestine. Urea can be easily hydrolyzed to ammonia and carbon dioxide by enzyme* urease*. So, elevated serum ammonia may result from its increased production, absorption (from intestine or urinary tract), decreased elimination of ammonia, and/or impaired ureagenesis. Hyperammonemia is always a sign of insufficient nitrogen excretion as discussed above, but it does not always necessitate symptomatic presentation. The signs and symptoms of hyperammonemia are usually neurological from mild cognitive and psychomotor changes to altered level of consciousness and coma [1]. Serum ammonia levels above 200 *μ*mol/L are reported to be associated with cerebral edema, herniation, and death [2].

It is imperative not only to diagnose early and manage efficiently but also to find the etiology of symptomatic hyperammonemia. Liver pathology, the most common cause of hyperammonemia, almost always presents with altered liver function tests. Hyperammonemia in the context of unremarkable (or normal) liver function tests becomes diagnostically challenging for physicians. The etiologies of nonhepatic hyperammonemia based on literature are summarized (Table 3). Hyperammonemia has been reported following lung and bone marrow transplantation, portosystemic shunts, ureterosigmoidostomy, chemotherapy of hematological malignancies, and solid organ tumors with 5-fluorouracil [3]. Infections with* urease*-producing bacteria such as* Proteus mirabilis*,* Helicobacter pylori*,* Cornebacterium*,* Klebsiella,* and* Morganella* species cause hyperammonemia presumably due to reabsorption of ammonia (generated from hydrolysis of urea) into the systemic circulation [4]. Hyperalimentation, valproic acid, and carnitine deficiency are reported to cause hyperammonemia [5–8]. In this patient, GI bleeding was initially considered for possible cause of hyperammonemia; however, serum ammonia level remained persistently high even after the treatment of GI bleeding. Elevated INR and aPTT were most likely secondary to vitamin K deficiency. Treatment with vitamin K improved the coagulopathy. All of the other above mentioned causes were essentially excluded with clinical history and relevant laboratory investigations.

Inborn errors in metabolism such as fatty acid oxidation defects, amino acid disorders, and urea cycle disorders that cause hyperammonemia are usually present early in childhood [9, 10]. Fatty acid oxidation defects are usually associated with hypoglycemic episodes [11], while amino acid disorders are with metabolic acidosis and ketosis [12, 13]. Both hypoglycemia and metabolic acidosis were not reported in this patient.

Urea cycle disorders (UCDs), as a result of deficiency of or defects in enzymes, usually present with hyperammonemia causing severe morbidity and mortality [14]. Literatures have shown a manifestation of late onset of one or more enzyme functional deficiency/ies unmasking the genetic disorders of the urea cycle in patients after bariatric surgery [15–18]. Markedly increased plasma glutamine, ornithine, and urinary orotic acid levels in the background of severe malnutrition and zinc deficiency may have led to unfolding of the functional deficiency of urea cycle enzyme/s leading to impaired ureagenesis and intractable hyperammonemia. Zinc deficiency can interfere with ornithine transcarbamylase (OTC) function [19, 20], which is possible in this patient. OTC deficiency, an X-linked disorder, is the most common inborn error of the urea cycle [21] and it usually has a fatal outcome soon after birth due to hyperammonemic coma. Heterozygous female may remain asymptomatic until the patient becomes acutely or chronically challenged by enough physiological stress. The recent gastrojejunal stent placement and decreased oral intake in chronically severe Protein-Energy Malnutrition (PEM) state probably led to increased physiological stress and catabolism in our patient. Blind loop syndrome with bacterial overgrowth in the patient with RYGBP (mainly distal type) may lead to illness, decreased oral intake, and increased catabolism. Alternative routes of enteral feeding such as gastrostomy may be needed to keep up with metabolism during an illness in such patients. In this case, no further information on the type of RYGBP was available as the procedure was done in another institution. DNA analysis of urea cycle enzymes would have given more definite etiology, but it was not carried out. Around 20–30% of patients with OTC deficiency are not detected in DNA analysis [22].

The initial goal of treatment should be to reduce ammonia production and absorption and facilitate elimination [23]. Intravenous glucose infusion should be started to provide a source of energy and raise insulin secretion that halts the protein breakdown due to its anabolic property. With the same token, protein intake should be restricted. IV lipid can also be given in increased energy demand. Alternative pathway therapies with sodium benzoate, sodium phenyl butyrate, and arginine have been proposed because these promote the synthesis of nitrogen-containing metabolites with high urinary excretion rates as an alternative to urea to remove waste nitrogen from the body [24]. In cases of uncontrolled hyperammonemia, hemodialysis may be an effective treatment. Due to rapid clinical deterioration and hemodynamic instability, hemodialysis could not be done in our patient. The mainstays of long-term management are dietary protein restriction, arginine or citrulline supplements, and oral alternative pathway medication to facilitate nitrogen excretion. Reversal of bariatric procedure is considered for the failure of weight loss or other complications due to the bariatric surgery itself. The decision of reversal tends to be highly individualized [25]. Consultation with an experienced bariatric surgeon may benefit when a patient presents with the nutritional/metabolic complication of bariatric surgery. This case report contains certain limitations such as being unable to perform DNA tests for urea cycle enzymes, hemodialysis, and use of aggressive scavengers such as sodium benzoate for management of hyperammonemia. Even with optimum therapy, this clinical entity is still associated with high rate of mortality.

In conclusion, hyperammonemia encephalopathy following bariatric surgery in the context of normal liver function tests becomes diagnostically challenging for physicians. The exact mechanism of hyperammonemia in such patient is still not clear but more data are gradually emerging in the support of cause-effect relationship among the triad of hyperammonemia, nutritional complications following bariatric surgery, and functional deficiency of urea cycle enzymes. We emphasize the importance of considering secondary causes of hyperammonemia in an adult woman after bariatric surgery. Early diagnosis and aggressive management are the only keys to improving survival.

## Figures and Tables

**Table 1:** Basic laboratory investigations.

Lab. test	Results	Ref. range
WBC	8.34	4.5–10.9 k/*μ*L
Hb	**5.4**	12.5–15.0 g/dL
Platelets	116	130–400 k/*μ*L
Sodium	136	135–145 mmol/L
Potassium	4.8	3.6–5.2 mmol/L
BUN	**11**	6–21 mg/dL
Creatinine	**2.1**	0.6–1.1 mg/dL
Glucose	113	70–140 mg/dL
INR	**2.15**	0.8–1.2
aPTT	**67.5**	28–38 sec
CPK	**466**	22–198 IU/L
AST	**49**	13–40 IU/L
ALT	**23**	17–35 IU/L
ALP	116	37–130 U/L
Albumin	**1.8**	3.5–5.0 g/dL
Prealbumin	**<5**	20–40 *μ*g/dL
HDL	**<5**	>50 mg/dL
LDL	95	<100 mg/dL
TG	133	<150 mg/dL
S. NH_3_^+^	**193**	<30 *μ*mol/L
P. zinc	**23**	60–130 *μ*g/dL
S. copper	57	70–175 *μ*g/dL
24 hr U. Cu	25	15–60 *μ*g/24 hr
Valproate	<1.0	50–100 *μ*g/mL
Lithium	<0.1	0.8–1.2 mmol/L

WBC: white blood cells; Hb: hemoglobin; BUN: blood urea nitrogen; PT: prothrombin time; INR: international normalized ratio; aPTT: activated partial thrombin time; CPK: creatinine phosphokinase; AST/ALT: aspartate/alanine aminotransferase; ALP: alkaline phosphatase; HDL: high-density lipoprotein; LDL: low-density lipoprotein; TG: triglycerides; P.: plasma; S.: serum.

**Table 2:** Special laboratory investigation panel.

Lab test	Results	Ref. range
*Plasma amino acid profiles:*		
Alanine	350	200–483 *μ*mol/L
Arginine	130	43–407 *μ*mol/L
Asparagine	131	31–64 *μ*mol/L
Aspartic acid	5	1–4 *μ*mol/L
Beta-alanine	3	<5 *μ*mol/L
Citrulline	43	16–51 *μ*mol/L
Glutamine	1363	428–747 *μ*mol/L
Glutamic acid	55	10–97 *μ*mol/L
Glycine	555	122–322 *μ*mol/L
Histidine	129	60–109 *μ*mol/L
Homocysteine	<1	<1 *μ*mol/L
Hydroxyproline	71	4–27 *μ*mol/L
Isoleucine	27	34–98 *μ*mol/L
Leucine	46	73–182 *μ*mol/L
Lysine	359	119–233 *μ*mol/L
Methionine	22	16–34 *μ*mol/L
Ornithine	149	27–83 *μ*mol/L
Phenylalanine	70	40–74 *μ*mol/L
Proline	632	104–383 *μ*mol/L
Serine	156	65–138 *μ*mol/L
Taurine	43	31–102 *μ*mol/L
Tryptophan	5	40–91 *μ*mol/L
Tyrosine	46	38–96 *μ*mol/L
Valine	73	132–313 *μ*mol/L

*Serum carnitine levels:*		
T. carnitine	88	31–67 nmol/mL
F. carnitine	61	25–55 nmol/mL

*Urine orotic acid level:*		
Ur. orotic acid	**2.3**	0.4–1.2 mmol/molcr

T.: total; F.: free; Ur.: urine.

**Table 3:** Differential diagnosis of nonhepatic hyperammonemia based on [3–8].

SN	Ddx	Characteristics
1	Medications	Valproic acid, 5-FU

2	Infections	Urease-producing bacteria:
		(i) *Proteus mirabilis*
		(ii) *Helicobacter pylori*
		(iii) *Cornebacterium*,* Klebsiella,* and *Morganella* species

3	Surgery	(i) Lung transplant
		(ii) Bone marrow transplant
		(iii) Ureterosigmoidoscopy
		(iv) Portosystemic shunts
		(v) Bariatric surgery

4	Hyperalimentation	Increased nitrogen load in patient receiving parental nutrition

5	Errors in metabolism	
	(a) Fatty acid oxidation defects	
	(b) Urea cycle enzyme defects	
	(c) Amino acid disorders	

6	Gastrointestinal bleeding	

7	Carnitine deficiency	

## Abbreviations

ABG: Arterial blood gas
ALT: Alanine aminotransferase
aPTT: Activated partial thromboplastin time
AST: Aspartate aminotransferase
BUN: Blood urea nitrogen
CT: Computed tomography
D5%: 5% dextrose
DNA: Deoxyribonucleic acid
ED: Emergency Department
EKG: Electrocardiogram
GCS: Glasgow Coma Scale
GI: Gastrointestinal
HDL: High-density lipoprotein
INR: International normalized ratio
IV: Intravenous
OTC: Ornithine transcarbamylase
PEM: Protein-Energy Malnutrition
PRBCs: Pack red blood cells
RYGBP: Roux-en-Y Gastric Bypass Procedure
SBP: Spontaneous bacterial peritonitis
UCD: Urea cycle disorder.
